# Determinants of collaboration dynamics and challenges in North-South data-driven health research in the African context: an epistemic injustice perspective

**DOI:** 10.3389/frma.2026.1885894

**Published:** 2026-09-10

**Authors:** Rahel Bekele, Anne Moen, Merga Belina, Kaija Saranto, Kiros Berhane

**Affiliations:** 1 School of Information Science, Addis Ababa University, Addis Ababa, Ethiopia; 2 Department of Public Health Sciences, University of Oslo, Oslo, Norway; 3 Department of Statistics, Addis Ababa University, Addis Ababa, Ethiopia; 4 Department of Health and Social Management, University of Eastern Finland, Kuopio, Finland; 5 Department of Biostatistics, Mailman School of Public Health, Columbia University, New York, NY, United States

**Keywords:** artificial intelligence, data science, epistemic injustice, equity in global health research, health research collaboration, human capacity in research partnership, North-South collaboration, North-South collaboration research practice

## Abstract

**Introduction:**

Collaborations between Global North and South institutions are increasingly important for advancing artificial intelligence (AI) and data science in healthcare, offering opportunities to address context-specific challenges and reduce inequities. However, these partnerships often face persistent issues related to sustainability, equity, and long-term impact. Drawing on concepts of epistemic injustice and structural imbalances between partners, this study examines the factors shaping the effectiveness of North–South data-driven health collaborations, with particular attention to which factors exert the strongest statistically significant influence on collaboration effectiveness.

**Methods:**

The study conducted a cross-sectional survey of participants from the Data Science Initiative for Africa (DS-I Africa) network, covering the entire continent of Africa, and their partners from the Global North, and applies structural equation modeling to assess key determinants of collaboration outcomes.

**Results:**

The analysis focuses on governance and leadership, human capacity, and project and institutional practices. Human capacity is the strongest positive predictor of collaboration effectiveness (β = 0.467, *p* = 0.008). Project and institutional practices show a significant negative association with collaboration effectiveness (β = −0.464, *p* = 0.016). Governance and leadership also demonstrate a significant effect on collaboration outcomes.

**Discussion:**

The findings demonstrate that sustainable and equitable North–South collaborations depend on the alignment of governance, human capacity, and institutional practices. Moreover, addressing epistemic injustice requires system-level changes that promote shared leadership, ethical data governance, and strengthening human capacity as a reciprocal and inclusive process central to achieving balanced knowledge production and long-term collaboration impact.

## 1 Introduction

In recent years, North–South (NS) collaborations between institutions in the Global North (from high-income countries) and the Global South (low- and middle-income countries) are uniquely positioned to accelerate collaborations and exchange capacity. The convergence of Data Science (DS) and Artificial Intelligence (AI) combines advanced computational and analytical expertise with contextual knowledge of local health systems, and this is an example where collaboration offers transformative opportunities for advancing global health research. The co-creation of innovative, locally relevant solutions can facilitate technology transfer, human capacity building, and institutional knowledge exchange, thereby strengthening research infrastructure in LMICs (Amann et al., 2022; Basu et al., 2017; Kok et al., 2017).

Application of these technologies enhances diagnostics, predictive modeling, health system optimization, and evidence-based policy-making, particularly when implemented through collaborative, cross-regional partnerships. Despite these advantages, the NS collaborations in data-driven health research can be challenging and uneven. There are several challenges to equitable NS collaboration. Asymmetries, structural inequities and persistent power imbalances reinforce dependency on Northern partners and can limit the long-term impact of collaborative initiatives. This imbalance can be related to factors such as funding priorities, research agendas, and project direction, since collaborations are most often initiated, managed, and sustained by employing leadership and governance structures common at Northern institutions (Bhakuni and Abimbola, 2021). This is limiting the agency of Southern partners to define locally meaningful objectives (Van der Veken et al., 2017; Rakotonarivo and Andriamihaja, 2023; Marjanovic et al., 2013). Data ownership and control remain another critical source of tension, where southern institutions often provide essential health data but lack equal control over its use, analysis, and dissemination, raising concerns regarding equity, intellectual property, and sustainability of research benefits (Munung et al., 2017). A further challenge is the disparity in human capacity, few funding opportunities to build necessary research and technical expertise, and constraints in advanced computational infrastructure, mentorship opportunities, and specialized skills needed, hindering many Southern institutions from fully engaging in Data Science and AI research (Matenga et al., 2019; Atkins et al., 2016). These are previously reported disparities that limit Southern partners' capacity to participate fully in setting research priorities, scaling solutions, and driving sustainable and equitable growth. Qualitative studies have pointed out social and cultural factors, including communication styles, misaligned norms, hierarchical perceptions, and historical legacies of colonialism, complicating collaboration dynamics, interpersonal relationships often leading to misunderstandings, eroding trust, and compromising partnership cohesion (Van der Veken et al., 2017; Gautier et al., 2018; Ward et al., 2018). Thus, leadership, an understanding of equitable project management, and the mediating and moderating roles of capacity building, cultural sensitivity, and collaboration are critical to establishing equitable, efficient, and sustainable NS partnerships in which benefits are shared equitably (French et al., 2024; Llanos et al., 2024).

Moreover, inadequate capacity building reduces equitable participation across all phases of research, from study design to implementation, analysis, and dissemination. Coupled with inconsistent project practices, including misaligned communication norms, decision-making procedures, and stakeholder engagement strategies, these limitations often lead to reduced efficiency and missed opportunities for mutual learning (Boaz et al., 2018; Semahegn et al., 2023). Additionally, insufficient attention to project transition and continuation mechanisms frequently results in the discontinuation of initiatives once funding ends, leaving minimal institutional memory or sustained benefits for local health systems (Kok et al., 2017). Moreover, resource constraints constitute yet another barrier. Northern partners generally have access to advanced computational infrastructure, long-term funding, and robust data storage systems, whereas Southern institutions often operate under significant financial and technological limitations (Umphrey et al., 2024; Nyangulu, 2023).

Drawing on reported literature and elaborating on the abovementioned challenges, and adopting a theoretical perspective of epistemic injustice Fricker, (2007), this study suggests potential solutions. Previous research report power asymmetries, inequitable funding, and authorship imbalances (French et al., 2024), the critical role of trust, appropriate policies and data sharing in NS collaborations and joint problem solving (Nezami et al., 2022) and importance of leadership experiences and participation in all phases of a R&D to enhance Southern institutions' autonomy, personal and institutional research capacity, and long-term sustainability of NS collaborations (Wao et al., 2021; Atkins et al., 2016). In her discussion of epistemic injustice, Fricker (2007) differentiates between testimonial and hermeneutic injustice. Testimonial injustice points to different perspectives about “what counts” or is seen as important to bring to a collaboration, and interpretation of hermeneutic injustice points to reasoning/interpretation of “what it means.”

French et al. (2024) examine equitable, transdisciplinary NS research when they propose a five-domain framework for responsible implementation: collaborative leadership, agile management, flexible consortia, researcher positionality, and co-design participation. This framework underscores reflexive, inclusive research management that builds Southern capacities and ensures mutual benefit. Nezami et al. (2022) highlight the importance of noting that siloed structures, fragmented data, and weak policies limit joint problem-solving. Wao et al. (2021) also investigated NS collaborations in HIV/AIDS clinical trials and found that, while such studies enhance research capacity in LMICs and focus on vulnerable populations, leadership and funding inequities persist. Atkins et al. (2016) demonstrate equitable partnership structures, capacity building, and collaborative e-learning through the ARCADE projects. Collectively, these studies reveal that the effectiveness of leadership in NS collaborations is shaped by a dynamic interplay of structural, relational, and contextual factors, emphasizing power dynamics, equitable participation, trust, and cultural awareness.

While these challenges are widely acknowledged in qualitative studies and anecdotal reports (Fricker, 2007; Gautier et al., 2018; Ward et al., 2018), there is limited empirical evidence quantifying factors affecting North-South Collaboration Outcomes. As such, empirical evidence quantifying the relative influence of leadership, data governance, human capacity, collaboration practices, and other socio-cultural factors on NS collaboration outcomes is limited, especially in leveraging equitable data science and AI advancements. Accordingly, it is essential to identify and understand the issues that must be addressed to improve the quality of collaboration and ensure that benefits are shared equitably among partners. Furthermore, determining which of these factors most strongly influence NS collaboration success is critical for designing partnerships that are equitable, efficient, and sustainable; identifying key areas for improvement and developing strategies that strengthen collaborative outcomes.

Accordingly, this study addresses the quantitative research gap by applying structural modeling to empirically examine the key enablers and barriers to successful NS collaborations in AI and DS for health. It investigates the research question: which factors exert the strongest statistically significant influence on collaboration success? Specifically, it attempts to address the question of how leadership factors influence collaboration success, the role of human capacity building in effectiveness and sustainability, the impact of project transition strategies and access to resources on continuity and scalability, how project practices, participation, and stakeholder engagement contribute to outcomes, how equitable data ownership affects collaboration results, and the ways in which social and cultural practices influence NS research dynamics. By addressing these questions, the study provides a comprehensive, evidence-based framework for guiding policymakers, funding agencies, and academic institutions in fostering balanced, contextually relevant, and sustainable NS collaborations in DS and AI research for health.

Drawing on key dimensions of epistemic injustice, the study elicits perspectives on key variables affecting NS collaboration: leadership, human capacity, project practices and transition strategies, data ownership and access to resources, and social-cultural practices, with mediating and moderating roles played by capacity building, cultural sensitivity, and equitable project management. The study also adds evidence-based guidance for NS partnerships that are inclusive and culturally informed to enhance global health research outcomes.

## 2 Theoretical framework and hypotheses development

The theoretical framework was initially developed based on the literature on factors influencing North–South research collaboration. Previous studies have identified a range of structural, institutional, and relational factors that shape the equity, effectiveness, and sustainability of North–South research partnerships. These include access to research resources, project practices, data ownership, governance and leadership, human capacity, project transition, and social and cultural practices. These seven constructs were initially treated as theoretically distinct because they represent different dimensions of the conditions under which North–South research collaborations are established, implemented, and sustained.

The framework is also informed by the perspective of epistemic (in)justice. From this perspective, inequalities in resources, authority, participation, knowledge, and decision-making opportunities are important because they can influence whose knowledge is recognized, whose perspectives are incorporated into research processes, who has control over research resources and data, and who is able to participate as an equal knowledge producer. The qualitative and conceptual literature therefore provides the substantive and theoretical basis for identifying the seven dimensions considered in the initial framework. However, the broad barriers documented in previous studies should be distinguished from the specific quantitative constructs evaluated in this study. The literature identifies challenges and mechanisms within North–South collaborations, whereas this study translates these dimensions into measurable survey-based constructs and examines their relationships quantitatively.

**Figure 1:**
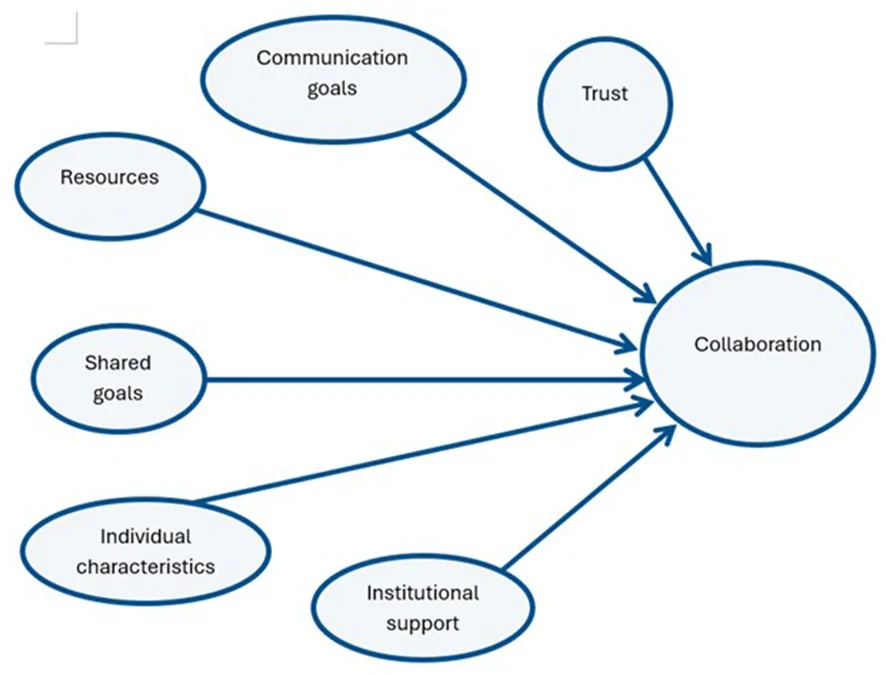
Initial theoretical framework of factors influencing North–South research collaboration.

The initial theoretical framework comprised seven constructs: access to research resources, project practices, data ownership, governance and leadership, human capacity, project transition, and social and cultural practices. Figure 1 presents the initial theoretical framework and the hypothesized relationships between these seven constructs and North–South research collaboration.

The seven constructs were initially considered theoretically distinct and informed the development of the survey instrument and the initial hypothesized relationships. The following sections provide the theoretical rationale for each construct and its expected relationship with North–South research collaboration.

### 2.1 Access to research resources

Access to research resources such as funding, infrastructure, technology, data, training, and research facilities is an important determinant of power dynamics and effectiveness in North–South collaborations. Bradley (2008) and Crane (2010) argue that persistent inequalities in resource availability can place Northern institutions in structurally dominant positions, influencing who sets research agendas, who leads projects, and who benefits from research outcomes. Studies have also highlighted that inadequate access to laboratory infrastructure, digital tools, and research funding can constrain Southern partners' ability to contribute as equal knowledge producers (Bezuidenhout et al., 2017). Conversely, equitable resource allocation through shared funding mechanisms, capacity building, and infrastructure strengthening can foster more balanced partnerships, enhance trust, and improve the long-term sustainability of North–South collaboration (Aellah et al., 2016).

From an epistemic justice perspective, unequal access to resources may constrain not only the material participation of Southern partners but also their ability to generate, contribute to, and influence knowledge. Thus, equitable access to research resources is expected to support more balanced and effective collaboration.

**H1: Equitable access to research resources positively affects North–South research collaboration**.

### 2.2 Project practices

Project practices in North–South collaborations refer to the operational norms, decision-making structures, role allocations, communication arrangements, and resource-management routines that guide collaborative research. These practices may reflect pre-existing asymmetries in funding, institutional capacity, and geopolitical power. Studies have shown that Northern partners may dominate agenda setting, leadership, and project governance, while Southern partners are assigned primarily implementation roles, resulting in limited ownership and marginal participation (Crane, 2010; Bezuidenhout et al., 2017). Such uneven practices can undermine trust, create dependency, and reinforce historical inequities. In contrast, collaborative practices that include joint proposal development, shared leadership, transparent communication, equitable role allocation, and co-creation of research outputs can enhance mutual respect, fairness, ownership, and long-term partnership sustainability (Aellah et al., 2016; Bradley, 2008).

From an epistemic justice perspective, project practices are important because they shape opportunities for Southern partners to participate in research decisions, contribute their knowledge, and exercise ownership over collaborative activities.

**H2: Equitable project practices positively affect North–South research collaboration**.

### 2.3 Data ownership

Aellah et al. (2016) discuss how unequal ownership of and access to health data in North–South research partnerships can erode trust and create ethical tensions. North–South collaborations may experience an uneven division of labor and control over data, reinforcing inequities in ownership. When Southern institutions have limited decision-making power over data governance, they may experience marginalization, which can foster mistrust and perceptions of exploitation. In contrast, equitable agreements that clearly define data ownership, access rights, governance responsibilities, and benefit sharing can create more trusting and balanced partnerships and strengthen Southern partners' willingness to participate fully in joint research.

From an epistemic justice perspective, data ownership is important because control over data is closely connected to control over knowledge production, interpretation, dissemination, and use. Equitable data governance can therefore strengthen the ability of Southern partners to participate meaningfully in knowledge production and benefit from research outputs.

**H3: Equitable data ownership and governance positively affect North–South research collaboration**.

### 2.4 Governance and leadership

Governance and leadership influence how authority, responsibility, and decision-making power are distributed between Northern and Southern partners. Studies report that when leadership is concentrated in Northern institutions through control of funding, agenda setting, authorship, and research priorities, Southern partners may experience reduced autonomy and limited influence over research decisions (Aellah et al., 2016; Bradley, 2008; Crane, 2010; Gaillard, 1994). Such imbalances can weaken commitment, generate perceptions of unfairness, and undermine long-term partnership sustainability.

In contrast, collaborative leadership models that distribute authority, encourage mutual accountability, promote joint decision-making, and recognize local expertise can support more equitable and productive North–South collaborations. Such approaches can strengthen ownership, enhance motivation, and support capacity development within Southern institutions. Governance and leadership are particularly relevant to epistemic justice because governance arrangements determine whose perspectives are heard, whose knowledge is recognized, and who has influence over research priorities and decisions.

**H4: Inclusive and collaborative governance and leadership positively affect North–South research collaboration**.

### 2.5 Human capacity

Human capacity is important for ensuring that Southern researchers can actively participate in, lead, and manage research projects rather than merely fulfilling subordinate or implementation roles. Maponga et al. (2023) indicate that developing human capacity within Southern institutions can strengthen the ability of local researchers to participate meaningfully in research activities and assume leadership responsibilities. Khisa et al. (2024) similarly argue that without comprehensive capacity-strengthening initiatives, Southern partners may remain dependent on Northern institutions for technical expertise, potentially perpetuating power imbalances and limiting the long-term sustainability of collaborations. Furthermore, Luthuli et al. (2024) report that when capacity building is not adequately embedded in collaborative projects, Southern partners may face difficulties in securing leadership positions or participating in decisions that influence research agendas.

Embedding capacity building through training, mentorship, infrastructure provision, and reciprocal knowledge sharing is therefore important for enhancing the autonomy and leadership of Southern institutions (Maponga et al., 2023; Khisa et al., 2024). From an epistemic justice perspective, human capacity is important because meaningful participation in knowledge production requires not only formal inclusion but also the skills, confidence, autonomy, and opportunities necessary to contribute and exercise influence.

**H5: Strengthened human capacity positively affects North–South research collaboration**.

### 2.6 Project transition

Effective project transition is important for ensuring that Southern partners gain the skills, knowledge, experience, and autonomy required to assume key roles and continue research activities after the project concludes. (Maponga et al. 2023) indicate that effective transition can contribute to the sustainability of collaborative research when Southern partners are adequately prepared to assume greater responsibility. However, when transitions occur abruptly or without sufficient preparation, they may result in loss of local ownership, reduced institutional capacity, and renewed dependency on Northern institutions (Khisa et al., 2024).

Recent studies indicate that gradual and structured transition processes involving mutual capacity building and shared leadership throughout the project can enhance the long-term sustainability and effectiveness of North–South collaborations (Luthuli et al., 2024). Effective transition strategies may include mentoring, joint decision-making, transfer of responsibilities, and clear agreements regarding the future roles of each partner. Such arrangements can strengthen institutional capacity, promote autonomy, and support continued collaboration after project funding ends.

**H6: Structured and equitable project transition strategies positively affect North–South research collaboration**.

### 2.7 Social and cultural practices

Social and cultural practices influence how leadership roles are assumed, decisions are made, communication occurs, and trust is negotiated within North–South collaborations. Rose et al. (2024), reflecting on experiences in African biomedical research collaborations, note that local social norms can directly influence leadership and decision-making processes. Longworth et al. (2024), in their systematic review of co-creation projects in low- and middle-income countries, identify relational connection and culture as important factors influencing implementation. Building trust across socio-cultural divides requires time, attention to local power relations, and culturally sensitive stakeholder engagement.

Without deliberate efforts to respect and integrate local social practices, including context-specific approaches to decision-making, communication, and community engagement, North–South collaborations may risk reinforcing existing power relations or alienating Southern partners. Conversely, cultural sensitivity and adaptive project practices can facilitate meaningful participation and contribute to fairer and more sustainable collaborations.

These practices are also relevant to epistemic justice because they influence whether local forms of knowledge, communication, and participation are recognized and incorporated into collaborative research processes.

**H7: Social and cultural sensitivity and the integration of local practices positively affect North–South research collaboration**.

These seven initial theoretical hypotheses provided the conceptual basis for developing and consolidating the constructs included in the final model.

## 3 Materials and methods

### 3.1 Study design and data collection

This study employed a cross-sectional survey design to examine the factors influencing North–South collaboration in data-driven healthcare research and development projects. Data were collected using a structured questionnaire consisting of closed-ended items measured on a five-point Likert scale, ranging from 1 (Strongly Disagree) to 5 (Strongly Agree).

The survey instrument was developed based on a review of relevant literature on North–South research collaboration and collaborative practices in health research (Lambert and Newman, 2023; Lam and Green, 2023). The constructs and measurement items were adapted from the literature and aligned with the objectives of this study.

To establish content validity, the initial questionnaire was reviewed by a panel of experts with extensive experience in data science and international collaborative research. The experts evaluated the relevance, clarity, comprehensiveness, and contextual appropriateness of the measurement items. Based on their feedback, revisions were made to improve item wording, content coverage, and construct representation. The revised instrument was subsequently pilot-tested with a small group of participants who are representative of the target population. This was done in order to assess clarity, comprehension, completion time, and the reliability of the measurement scales. Minor refinements identified during the pilot phase were incorporated before the questionnaire was finalized for data collection.

The final questionnaire consisted of two sections. The first section collected demographic information, including institutional affiliation, educational level, organizational role, and project involvement. The second section measured the study constructs related to collaborative practices in North–South healthcare research partnerships.

Each construct was operationalized as a latent variable (see Table 1) measured using multiple reflective indicators. The measurement items were designed to capture respondents' experiences and perceptions of collaborative practices in data-driven healthcare research. The study examined seven key dimensions influencing North–South collaboration (5 items), Leadership (5 items), Data Ownership (4 items), Human Capacity (3 items), Project Practices (4 items), Transition and Continuation (4 items), Access to Resources (3 items), and Social and Cultural Practices (3 items). All constructs were measured using multiple Likert-scale items, with higher scores indicating stronger perceptions of effective collaborative practices.

**Table 1:** Operationalization of the study constructs.

Study construct	Operational definition
North–South collaboration	The extent to which partners from higher-income (“Northern”) and lower-income (“Southern”) institutions jointly design, implement, and co-author work, with roles and influence distributed rather than concentrated in one partner.
Leadership	Who holds decision-making authority over project direction, funding allocation, and strategic priorities—measured by whether Southern partners hold PI/co-PI roles or are confined to implementation/advisory roles.
Data ownership	Who controls collection, storage, access, and rights to use, publish, or share study data including whether data generated in-country remains under local institutional custodianship.
Human capacity	The degree to which the collaboration builds durable skills, credentials, or expertise among local staff/researchers (training, mentorship, degree support) vs. relying on short-term external expertise.
Project practices	The day-to-day operational norms of the collaboration communication channels, meeting practices, budget transparency, and whether procedures reflect shared input or are set unilaterally.
Transition and Continuation	Plans and mechanisms for sustaining project activities, infrastructure, or outcomes after external funding or partner involvement ends, including handover of systems and responsibilities to local actors.
Access to resources	The distribution of funding, equipment, technology, and infrastructure between partners, and whether Southern partners have direct (not pass-through) access to resources needed for their role.
Social and cultural practices	The degree to which the collaboration respects, incorporates, and adapts to local norms, languages, and ways of working, rather than imposing external institutional or cultural defaults.

Data were collected during the Data Science for Health Discovery and Innovation in Africa (DS-I Africa) Consortium Conference, held in Ghana in August 2025. Participants were recruited from the DS-I Africa network, which provides a comprehensive representation of contemporary North–South collaborations in African health data science research. The network comprises 38 collaborative projects involving institutions across Africa and partners from the Global North, covering research, research training, ethics and social implications, capacity building, and consortium coordination activities.

The breadth of projects within the DS-I Africa network enabled the inclusion of participants representing different countries, institutions, disciplines, and partnership models, making it an appropriate setting for examining the characteristics and dynamics of North–South collaboration in health data science. Consequently, the participants were considered representative of the study context, particularly within Africa, as the conference brought together researchers and professionals from both Northern and Southern institutions actively engaged in data science and AI-based health research. Although the study focused primarily on African collaborations, many participants were involved in broader international partnerships; therefore, the findings may also have relevance to North–South collaborations in other Global South settings.

The complete survey instrument is provided as Supplementary material.

A structural equation modeling (SEM) approach was used in the analysis. Hair and Alamer (2022) note that SEM is particularly well suited for analyzing complex models, especially when the primary objective is prediction. They further highlight that SEM is advantageous when the dataset does not meet the assumption of normality and when the available sample size is relatively small. In this study, SEM was applied to examine relationships among constructs measured using multiple observed indicators derived from survey items. The initial measurement model included seven exogenous latent constructs: Leadership, Data Ownership, Human Capacity, Project Practice, Project Transition, Access to Resources, and Socio-Cultural Context and one endogenous latent construct representing North–South Collaboration. Specifically, the study assessed key dimensions of North–South collaboration, including leadership, human capacity, project practices, access to resources, data ownership, and socio-cultural factors, using a structured survey instrument.

Consistent with SEM best practices, constructs were expected to be measured by at least two indicators. Constructs with only one indicator or weak measurement properties were considered unstable and subject to removal during model refinement.

Structural equation modeling was conducted using the “lavaan” package in R (version 4.5.2). Given the ordinal nature of the survey items, all indicators were treated as ordered categorical variables, and models were estimated using the Full Information Maximum Likelihood (FIML) approach. The Maximum Likelihood with Robust Standard Errors (MLR**)** estimation technique was used to allow for robust estimation with Huber-White's robust standard errors.

Model evaluation followed established guidelines and included both absolute and incremental fit indices, namely the Comparative Fit Index (CFI), Tucker–Lewis Index (TLI), Root Mean Square Error of Approximation (RMSEA), and Standardized Root Mean Square Residual (SRMR).

Finally, based on the above-described model-building process, conceptually related constructs will be grouped into higher-order latent variables to further reduce model complexity via parsimony.

### 3.2 Model building and fit assessment

The model building process started with simultaneous consideration of all seven exogenous latent variables in the SEM model, followed by a careful process described below under model refinement and simplification to assess model fit in order to arrive at the best-fitting but most parsimonious final model.

To address identification and stability issues, a stepwise model refinement strategy was implemented:

**(i) Model simplification -** The number of predictors was reduced, retaining only theoretically central and well-measured constructs.
**(ii) Removal of weak constructs -** Latent variables with fewer than two viable indicators (e.g., Access to Resources) or evidence of unstable estimation (e.g., with negative residual variances) were removed.
**(iii) Item selection -** Indicators with low standardized factor loadings or problematic residuals were excluded. Only items with acceptable standardized loadings (≥ 0.50) and no estimation anomalies were retained.
**(iv) Measurement model verification -** Measurement models were assessed before estimating structural relationships to ensure adequate construct validity.

### 3.3 Final SEM specification

The final SEM model was specified through an iterative model-building process that considered model convergence, theoretical relevance, and the need for construct parsimony given the modest sample size. Due to data sparsity resulting from the limited number of observations, related constructs were consolidated based on their conceptual alignment with the study objectives. This approach is consistent with SEM practices for studies with relatively small samples, where reducing model complexity can improve estimation stability and interpretability. The consolidated model was subsequently evaluated to determine whether increased parsimony resulted in more robust and meaningful relationships among the study constructs.

### 3.4 Model evaluation and reporting

Final model fit was assessed using multiple fit indices. Models demonstrating acceptable convergence, reasonable fit indices, and interpretable parameter estimates were retained for interpretation. Model specification decisions were guided by both statistical criteria and theoretical justification, avoiding purely data-driven modifications. As mentioned before, all analyses were conducted in R (version 4.5.2) using the “lavaan,” semPower, and semPlot packages.

Overall, the modeling strategy prioritized parsimony, stability, and interpretability, given the modest sample size and ordinal nature of the data. Overly complex models were avoided to reduce the risk of non-identification and unreliable parameter estimates. This approach ensured that the final SEM results were statistically valid and theoretically meaningful. Finally, we have also conducted a power analysis for the SEM model. The results, *post-hoc* power analysis (~93%), show that the sample size is adequate to fit the SEM model.

## 4 Results

The profile of respondents provides an overview of the demographic and professional characteristics of the participants included in this study. This section presents relevant information such as academic rank, project affiliation, and other pertinent variables that help contextualize the data gathered and ensure a better understanding of the composition of the respondents.

As mentioned before, among our 109 respondents, 79 (72.5 %, *N* = 109) represented “Primarily Global South,” and 30 (27.5%, *N* = 109) represented “Primarily Global North.” Table 2 summarizes the distribution of respondents according to the key profile variables. It should be noted that, as some categories include multiple-choice questions, the totals may exceed the number of respondents because of double-counting.

**Table 2:** Profiles of respondents.

Variables	*N* = 109^a^
Primary institutional affiliation	
Primarily Global North	30 (28%)
Primarily Global South	79 (72%)
Role in the collaboration (multiple categories allowed)	
Project leader/Principal coordinator	37 (28.7%)
Co-investigator	36 (27.9%)
Data manager/Analyst	17 (13.2%)
Junior researcher/Early career researcher	21 (16.3%)
Post-doctoral researcher	6 (4.7%)
Other	12 (9.3%)
Type of collaboration (multiple categories allowed)	
Research project	77 (50.7%)
Development/Implementation project	26 (17.1%)
Capacity building/training	42 (27.6%)
Other	7 (4.6%)
Years of experience in collaborative projects	
Less than a year	5 (4.6%)
1 to 2 years	17 (16%)
3 to 5 years	31 (28%)
More than 5 years	56 (51%)
Number of North–South projects involved in	
Just 1	37 (34%)
2–4	47 (43%)
More than 4	25 (23%)
Highest level of education	
Doctoral	64 (59%)
Masters	35 (32%)
Bachelors	7 (6.4%)
Other	3 (2.8%)
Academic rank	
Professor	20 (18%)
Associate professor	21 (19%)
Assistant professor	20 (18%)
Lecturer	21 (19%)
Not in academia	27 (25%)

^a^The total sample size of study participants from which the percentages are calculated.

Based on the cross-sectional design, a total of 109 respondents filled out the online survey. After data cleaning, 107 cases were retained for analysis. This sample size was considered modest for structural equation modeling (SEM), necessitating careful attention to model parsimony and identification.

An initial SEM was specified, including all seven exogenous latent variables simultaneously predicting North–South Collaboration, with correlated exogenous factors. However, this model exhibited poor statistical performance (as shown in Table 3 below), including non-convergence, missing standard errors, negative residual variances, and poor absolute fit indices.

**Table 3:** Model estimates for the initial model.

Model performance indices	Values
Robust Comparative Fit Index (CFI)	0.334
Robust Tucker-Lewis Index (TLI)	0.285

These issues were attributed primarily to excessive model complexity relative to the sample size, weakly measured constructs (e.g., latent variables with one or two indicators), and high collinearity among predictors. Given these limitations, the initial model was deemed statistically unstable and unsuitable for interpretation.

Following non-convergence issues with the complex SEM model with simultaneous inclusion of all seven constructs, which didn't converge, we redefined some constructs. As a theoretically informed alternative, conceptually related constructs were grouped into higher-order latent variables to further reduce model complexity. Specifically, first-order constructs were combined into three higher-order domains: Governance and Leadership, Human Capacity, and Project & Institutional Practice. This approach reduced the number of regression paths and parameters while preserving the theoretical structure of the model.

**Figure 2:**
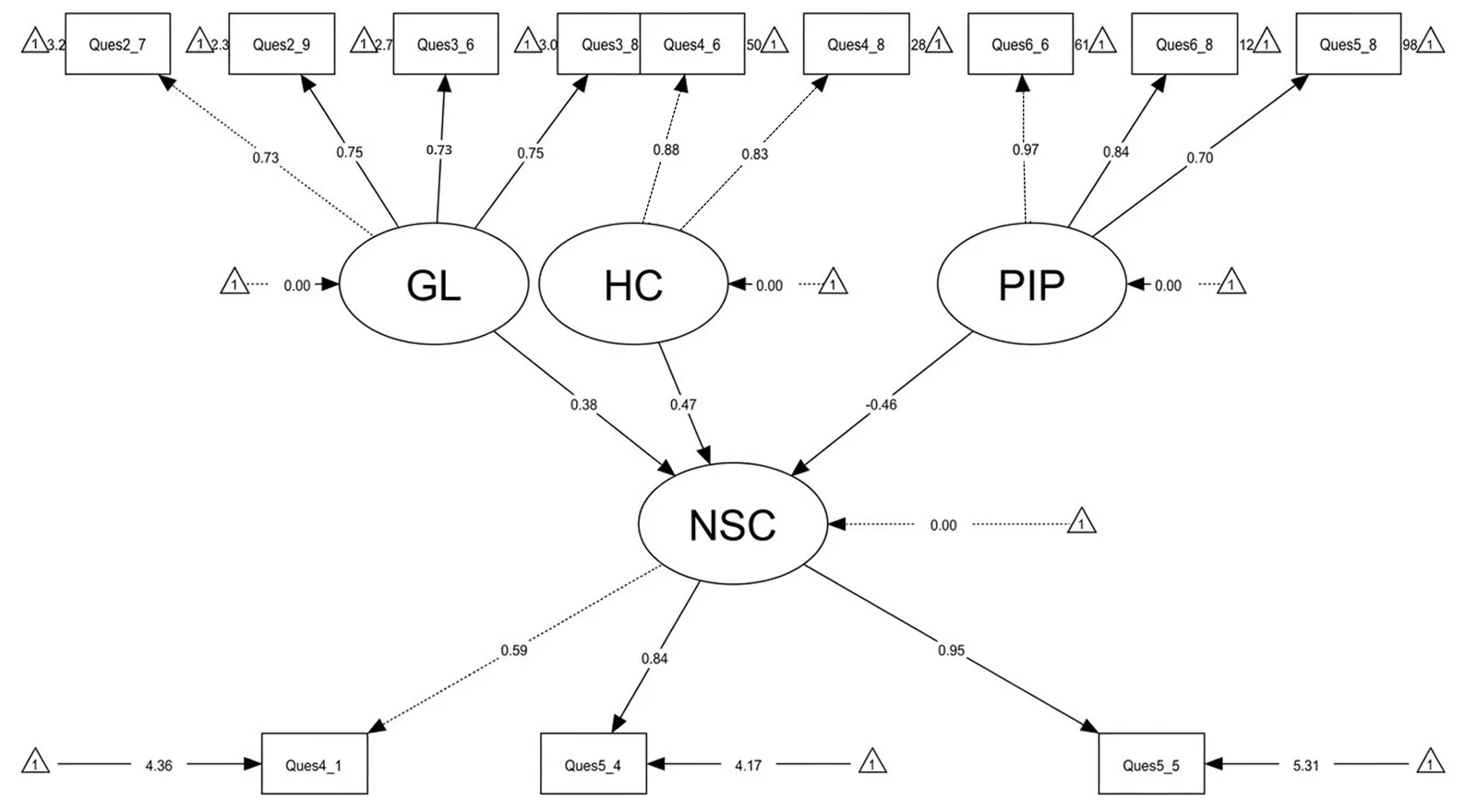
SEM diagram for the final model.

The final model retained three exogenous latent constructs: Governance and Leadership, Human Capacity, and Project & Institutional Practice and one endogenous construct—NS Collaboration. Each retained latent variable was measured by at least two indicators, ensuring proper model identification. We considered the three latent variables for the SEM model after running CFA for each of them, where the fit was excellent (Figure 2 and Table 4). Descriptive statistics for all items contributing to the three latent constructs in the final structural model are presented in Table 5.

**Table 4:** Model estimates for the final model.

Latent variable	Estimate	S.E.	*z*	*p*		LLCI	ULCI	Beta
Governance leadership	0.281	(0.139)	2.029	0.042	^*^	0.010	0.553	0.381
Human capacity	0.335	(0.127)	2.646	0.008	^**^	0.087	0.583	0.467
Project & institutional practice	−0.284	(0.117)	−2.417	0.016	^*^	−0.513	−0.054	−0.464

^*^p < 0.05, ^**^p < 0.01.

**Table 5:** Descriptive statistics of key factors influencing North–South collaboration.

North South collaboration	Mean	Median	SD
• Building human capacity in the Global South supports the recognition of local knowledge and expertise.	4.3	5	0.98
• Partners from the Global South are actively involved in shaping research questions and methodologies	3.9	4	0.95
• The project fosters co-production of knowledge	4.2	4	0.79
Governance & leadership			
• Partners from the Global North dominate in defining research questions, processes, and outputs	3.5	3	1.08
• Lack of ethical collaboration	2.8	3	1.18
• Lack of clarity on data ownership at project start	3.3	3	1.17
• Power imbalances leading to Global North dominance in data decisions	3.6	4	1.16
Human capacity			
• Limited funding for AI/Data Science training activities	4.1	4	0.94
• Unequal access to training resources	4.2	4	0.97
Project & institutional practice			
• Insufficient mechanisms for conflict resolution	3.5	3	1.00
• Power imbalances affecting who controls the transition process	3.4	3	1.11
• Poor communication between partners during transitions	3.2	3	1.08

### 4.1 Descriptive statistics of key factors influencing North–South collaboration

The structural component of the model specified direct paths from Governance and Leadership, Human Capacity, and Project & Institutional Practice to North–South Collaboration. This parsimonious specification substantially reduced the number of estimated parameters and improved model stability.

As a sensitivity analysis, we fitted the SEM on only the participants from the Global South.

## 5 Discussion

This study examined determinants of North–South Collaboration (NSC) through a parsimonious structural model comprising Governance and Leadership (GL), Human Capacity (HC), and Project & Institutional Practice (PIP). Interpreting these findings through the lens of **epistemic (in)justice** provides deeper insight into how collaboration dynamics either challenge or reproduce global inequities in knowledge production.

One important feature of the study is the imbalance between Global South and Global North respondents, with a significant majority (72.5%) based within the African continent. This was a reflection of the composition of investigators in the Data Science Initiative for Africa (DS-I Africa) network that was the sampling base for our survey. This distribution also likely reflects actual participation patterns in collaborations since sub-teams from the Global South tend to be bigger compared to collaborators from the Global North. Our modeling approach included sensitivity analysis on the Global South respondents only (the only group large enough to allow the modeling) to show that the main findings did not change from those with the whole data set. While impossible to rule out completely, the sensitivity analysis provides evidence against the potential impact of response bias.

An important advantage of using the DS-I Africa network as a sampling base was its likelihood of including almost all of the major players in the field and covering the entire African continent across the 38 projects within the network. The respondents from the Global North (while almost exclusively from the United States of America) are seasoned investigators who have been immersed in North-South collaborations and are hence well-positioned to provide valuable insights.

The predominance of respondents from the Global South (72.5%) offers an important corrective to the historical underrepresentation of Southern perspectives in research on international collaboration. From an epistemic justice standpoint, this strengthens the study by amplifying voices that are often marginalized in global knowledge systems. As such, the survey brings perspectives to human capacity building, adding new/additional insights surfacing in the answers to the questions (testimonial aspects of epistemic injustice), and the reported findings (hermeneutic aspects of epistemic injustice). However, the imbalance relative to Global North respondents (27.5%) may limit the extent to which power holders' perspectives are captured. This asymmetry reflects broader structural dynamics in which collaborative knowledge is often produced without adequately incorporating those most affected by inequities.

As shown in Table 2, the academic roles of respondents are almost evenly spread across academic ranks. Reflective of the nature of the DS-I Africa sampling base, 56% are project leaders or co-investigators, while a relatively smaller proportion are junior researchers and data staff. Given the nature of the survey, the composition of the sample is quite appropriate. Role descriptions are based on respondents' own accounts of their organizational responsibilities. They are not intended to imply hierarchical dynamics or limited engagement with junior colleagues. The study did not assess internal team interactions or supervisory practices.

The concentration of respondents in senior roles, including project leaders and co-investigators, provides valuable insight into governance processes and leadership challenges. Our findings may reflect hierarchical knowledge structures where epistemic injustice can arise if decision-making authority is concentrated among a limited group, potentially sidelining the experiential knowledge of junior researchers and implementers. The positive and significant effect of Governance and Leadership (β = 0.381, *p* = 0.042) suggests, however, that inclusive and collaborative leadership can mitigate such injustices, enabling capacity building by broader participation in knowledge production and decision-making.

In our study, human capacity emerged as the strongest positive predictor of NSC (β = 0.467, *p* = 0.008), and this underscores the central role of the participants to enable equitable participation. Available funding opportunities to build and maintain research capacity and technical expertise to manage the advanced computational infrastructure are necessary for Data Science and AI advancement. Evolving human capacity and funded opportunities to obtain necessary knowledge and skills are important to secure conditions for equitable engagement in DS and AI research (Matenga et al., 2019; Atkins et al., 2016). The importance of human capacity to drive epistemic justice, ensuring that the perspectives of participants from the South and North are articulated and acknowledged in data collection and interpretation, is important for productive collaboration to shape research priorities as well as sustainable and equitable growth. Disparities in training, skills, access to research resources and opportunities to participate in all tasks are key drivers of epistemic injustice (Bhakuni and Abimbola, 2021). Strengthening human capacity is therefore an encouraging finding, particularly within Global South institutions, as this enhances the ability of and opportunities for researchers to contribute meaningfully to knowledge production. Being vigilant to the continued influence of historical legacies of colonialism, power asymmetries, as well as the impact of social and cultural factors, like hierarchical perceptions, norms or communication styles, is important, as previous studies suggest that epistemic injustice thrives on misunderstandings, unproductive collaboration, and eroding trust in interpersonal relationships (Gautier et al., 2018). Equally important in terms of mutual benefits advancing Data Science and AI use in the Global South is human capacity and collaboration. To enhance Southern institutions' autonomy, the model's emphasis on Project & Institutional practices points to going beyond unidirectional knowledge transfer toward sustainable, reciprocal exchange, recognizing the value of testimonial dimensions of epistemic injustice, and exemplifying opportunities to include locally grounded expertise and diverse epistemologies (Wao et al., 2021; Atkins et al., 2016).

In Section 4, we report that Project and Institutional Practice was negatively associated with NSC (β = −0.464, *p* = 0.016), suggesting that current structures may constrain equitable collaboration. This finding is particularly significant when viewed through the lens of epistemic injustice: testimonial as well as hermeneutic injustice. Testimonial injustice points to different perspectives about “what counts” or is seen as important to bring to a collaboration, and hermeneutic injustice points to reasoning/interpretation of “what does it mean” (Fricker, 2007). Attention to project practices and specifically transition and continuation mechanisms once available funding ends are important to build institutional memory and sustained benefits for local health systems (Kok et al., 2017). Control over what constitutes valid data and novel research outputs is a prominent challenge in project work and joint problem solving, requiring project and institutional practices that ensure trust and appropriate policies for data sharing in NS collaborations (Nezami et al., 2022). The institutional and project practices often determine whose interpretations are validated and disseminated and are important for equitable contributions and critical for epistemic power. Furthermore, inequities in resource allocation, authorship, and data ownership can systematically privilege certain actors while marginalizing others. Northern partners generally have long-term funding for advanced computational infrastructure and robust data storage systems, whereas Southern institutions lack such institutional capabilities since they often operate under significant financial and technological limitations (Umphrey et al., 2024; Nyangulu, 2023).

The predominance of research-oriented collaborations (50.7%) shapes the study's epistemic context. Research collaborations often prioritize academic outputs, which may privilege certain forms of knowledge such as quantitative or Western scientific approaches over others. The results of our study therefore indicate that without deliberate attention to testimonial and interpretive aspects in knowledge production, efforts to ensure fairness and transparency, institutional and project-level practices may reinforce existing epistemic hierarchies that are often detrimental to fully leverage opportunities to advance Data Science and AI use in the Global South. Southern institutions may be actively engaged in research projects and provide data, but lack equal control over their data use, analysis, or dissemination (Bhakuni and Abimbola, 2021). This contributes to epistemic injustice by marginalizing some knowledge systems, including community-based or indigenous perspectives. Expanding the scope of collaboration to more inclusive, practice-oriented models may help address these imbalances.

Moreover, governance and capacity dynamics differ significantly between research and implementation settings, further jeopardizing epistemic justice. Structural limitations in long-term financial resources and advanced technological infrastructure in Southern institutions should be acknowledged when interpreting the effectiveness of North–South collaborations and assessing epistemic justice.

Below are also some methodological constraints that need to be discussed in some detail.

### 5.1 Initial SEM problems

We started fitting the model with seven constructs, but due to the small sample size, we got very poor model fit indices. Consequently, we reduced the number of constructs to three based on grouping concepts that analytically share important commonalities. The challenges encountered in the initial SEM model, including non-convergence and poor fit, highlight the limitations of applying complex statistical models to modest sample sizes. From an epistemic perspective, methodological constraints can influence which forms of knowledge are considered valid or robust. Overly complex models that fail to converge may obscure rather than illuminate meaningful relationships, underscoring the importance of methodological choices in shaping knowledge production.

Penalized likelihood methods have been developed in the statistical literature and are particularly useful when the sample size is small relative to the number of variables in the model (Rosseel, 2020; Hastie et al., 2009). In small-sample Structural Equation Modeling (SEM) studies, one of the biggest challenges is the rapid drop in model convergence reliability and parameter estimate stability when the sample size goes below traditional thresholds, such as fewer than 200 cases. This often results in solutions that don't work, like negative variances or non-positive definite covariance matrices. The main takeaway from this limitation is that statistical power and model complexity are inversely related in small samples. Trying to use a complex model with many parameters while lacking sufficient data almost guarantees problems with estimation. Overly complex models often fail to converge, while well-defined, low-parameter models' use of penalized likelihood methods can provide stable, interpretable results even with sample sizes under 100. Therefore, small-sample SEM is not inherently flawed, but it requires careful theoretical refinement and a conscious trade-off between the scope of analysis and reliability. In this study, Full Information Maximum Likelihood (FIML) estimation was used because it performs well under MCAR and MAR missing-data conditions (Cham et al., 2017).

### 5.2 Justification of model simplification

In this study, as highlighted earlier, in the beginning we started with seven constructs and the model resulted in very poor fit indices. We then employed theory-driven aggregation of constructs to streamline the analytical framework and reduce the number of variables. Guided by established theoretical models, related indicators and subscales were conceptually grouped and combined into higher-order composite constructs. This approach not only minimized redundancy while preserving theoretical integrity but also enhanced interpretability and statistical power by focusing on broader, more parsimonious dimensions without sacrificing substantive meaning. The constructs are simplified to three higher-order constructs: Governance & Leadership (GL), Human Capacity (HC) and Project & Institutional Practice (PIP).

The decision to group constructs into three higher-order domains (GL, HC, PIP) reflects a theoretically informed effort to balance complexity and interpretability. This simplification not only improved statistical performance but also aligns with a systems-oriented understanding of collaboration. From an epistemic justice standpoint, such aggregation can help foreground broader structural dynamics rather than fragmenting them into isolated variables, thereby supporting more holistic interpretations of inequality. However, prioritizing higher-order domains may capture shared variance while obscuring unique effects associated with lower-order constructs. Thus, the present model does not allow the distinct residual variance or effects of lower-order factors to be examined separately. In our context, although the consideration of higher-order factors was also consistent with the constraints of the available sample size, this was not the sole basis for aggregation. Rather, we prioritized the theoretical meaningfulness of the higher-order domains and took this limitation into account when interpreting the results.

Higher-order factor aggregation offers advantages in parsimony, reduced multicollinearity, and greater bandwidth. However, this comes at a cost: the higher-order factor primarily captures shared variance, while unique residual variance specific to each lower-order construct is relegated to residuals or averaged away. As a result, distinct predictive patterns, differential relationships with outcomes, and facet-specific effects can be masked, leading to loss of precision and potential misattribution of mechanisms. This limitation is particularly relevant for multidimensional constructs such as data ownership (e.g., different accountability or control arrangements) or transition strategies (e.g., phased vs. big-bang approaches). Although these facets may correlate and support a higher-order model, collapsing them can obscure unique effects. However, with the sample size we have available, collapsing the constructs in ways that do not unduly undermine our main objectives was considered to be a prudent and responsible approach. As a result, we claim the final model is more stable and appropriately parsimonious.

### 5.3 Validity and stability of final model

The final model demonstrated good fit and stability, with all three domains showing statistically significant relationships with NS Collaboration. This supports the validity of the conceptual framework and suggests that governance, capacity, and institutional practices are key dimensions of collaboration. The ability to identify these relationships empirically contributes to making visible the structural factors that underpin epistemic (in)justice in collaborative research.

### 5.4 Sensitivity analysis (Global South)

The robustness of the findings was assessed via thorough sensitivity analyses. Results remained consistent when only Global South participants were analyzed, highlighting that the model reflects Global South realities (see Table 6). Although the figures are not the same, the model estimates from the Global South-only data are almost similar to the final model, except that one construct (Governance and Leadership) was not significant at a 5% level of significance.

**Table 6:** Model estimates using the Global South data only.

Latent variables	Estimate	S.E.	*z*	*p*		LLCI	ULCI	Beta
Governance leadership	0.306	(0.178)	1.722	0.085	.	−0.042	0.654	0.405
Human capacity	0.369	(0.145)	2.539	0.011	^*^	0.084	0.653	0.488
Project & institutional practice	−0.239	(0.117)	−2.034	0.042	^*^	−0.469	−0.009	−0.403

^*^Significant at 5% level of significance, ^.^ Significant at 10% level of significance.

The consistency of results in the Global South–only analysis reinforces the robustness of the findings and highlights their relevance to contexts where epistemic injustice is most pronounced. This suggests that the identified dynamics are not driven solely by Global North perspectives but reflect lived experiences within Global South institutions. It also underscores the importance of centering these perspectives in future research.

Taken together, the findings suggest that addressing epistemic injustice requires **c**oordinated action across governance, human capacity, and institutional practice. Strong governance structures are essential for enabling inclusive leadership, accountability, and decision-making authority that genuinely creates space for diverse forms of knowledge. Adequate human capacity, supported through sustained capacity strengthening, allows partners to participate equitably, exercise agency, and influence research agendas. At the same time, supportive institutional practices including reforms to funding arrangements, authorship norms, and project management must be put in place to ensure a fair distribution of resources, recognition, and responsibility.

Without deliberate attention to all three domains, collaborations risk reproducing the very inequalities they seek to address. This underscores the need to move beyond isolated, project-level interventions toward active engagement with the governance, capacities, and institutional conditions shaping research partnerships. Such an approach is more likely to generate durable, system-level improvements in NS collaboration.

## 6 Conclusion and future research directions

The Research findings, derived from the SEM analysis, indicate that leadership, project practice, and human capacity are the key dimensions shaping North–South collaboration; when interpreted through the lens of epistemic injustice, these dimensions also reveal how inequalities in knowledge recognition and meaning-making persist. In relation to leadership, the results point to the need for more shared and inclusive leadership structures that ensure Southern partners are not only represented but have real authority in decision-making. This is particularly important for *testimonial injustice*, as it directly influences what counts as credible data and knowledge within collaborations. When leadership is concentrated in Northern institutions, local perspectives risk being undervalued or dismissed; therefore, emphasizing shared leadership helps redistribute epistemic authority and validates diverse forms of knowledge, including experiential and context-specific insights.

The findings on project practice further suggest that collaboration outcomes depend heavily on how knowledge is produced, interpreted, and applied in context. From an epistemic injustice perspective, this highlights the importance of promoting contextually relevant and sustainable practices that go beyond standardized or externally imposed models. Here, *hermeneutical injustice* becomes particularly salient, as reliance on dominant frameworks can limit the ability of Southern partners to articulate findings and realities on their own terms. Addressing this requires embedding interpretive flexibility into project design, ensuring that methodologies, evaluation criteria, and implementation strategies are grounded in local contexts. This also connects to the need for ethical data practices and responsible project transition, where data ownership, analysis, and long-term use are shared equitably, and where projects leave behind lasting value rather than extractive outcomes.

Finally, the strong influence of human capacity in the SEM results underscores the need to rethink capacity building as a core element toward epistemic justice. Rather than treating capacity as a deficit to be filled, the findings support a shift toward mutual and context-sensitive human capacity development, where all partners are recognized as knowledge holders. This approach helps counter testimonial injustice by affirming the credibility of Southern expertise, while also addressing hermeneutical injustice by expanding the range of knowledge systems and interpretive frameworks that inform collaboration. Investing in long-term capacity through skills development, institutional strengthening, and equitable access to research and publication opportunities ensures that knowledge production is not only shared but also sustained beyond the lifespan of individual projects.

Overall, the results suggest that embedding epistemic justice within leadership, project practice, and human capacity is essential for achieving more equitable NS collaboration. Practically, implementing strategies to address structural challenges and promote equitable international collaborations include setting up (1) joint leadership committees with shared decision-making authority, (2) equitable data governance agreements that define data ownership, access, and benefit-sharing before data collection begins, (3) allocating dedicated funding for reciprocal capacity building, for example as researcher exchanges and co-supervision of students, and (4) mechanisms for jointly evaluating partnership outcomes. These strategies improve the practical applicability of our findings to develop international health, data science and AI collaborations. Coupling this with practicing shared leadership, emphasizing contextually grounded practices, ethical data governance, and inclusive capacity development provides a pathway for addressing *testimonial* and *hermeneutical* injustice, ultimately enabling more balanced and co-produced knowledge systems.

Beyond these specific strategies, the findings have broader implications for stakeholders involved in AI-based health research collaborations, including funding agencies, research institutions, universities, and policymakers. As data-driven and AI-driven health research increasingly shapes global health innovation, strengthening North–South cooperation requires moving beyond short-term project-based partnerships toward sustainable and equitable research ecosystems. Funding bodies should encourage collaborative models that prioritize shared leadership, fair resource allocation, responsible data governance, and long-term capacity strengthening. Research institutions and universities should promote institutional partnerships, equitable authorship and knowledge-sharing practices, and opportunities for joint research leadership. Policymakers should support frameworks that ensure ethical AI development, protect equitable data ownership and access, and recognize the contributions of researchers and communities in the Global South. These measures can help address persistent structural inequalities in knowledge production and promote more inclusive, sustainable, and mutually beneficial Data-Driven and AI-based health collaborations.

What is more, North–South Data-Driven health collaborations should incorporate clear agreements on financial responsibilities, authorship and intellectual contributions, and data governance at the beginning of project development. Equitable financial models should ensure that Southern partners have adequate resources to participate meaningfully throughout the research lifecycle rather than serving primarily as data collection sites. Similarly, authorship policies should be established transparently and aligned with actual contributions, ensuring recognition of intellectual leadership from all collaborating partners. Data-sharing arrangements should also be defined collaboratively, addressing ownership, access rights, analysis responsibilities, benefit-sharing, and long-term stewardship to promote ethical and sustainable knowledge production.

The current study identifies leadership & governance, human capacity, and project & institutional practice as key factors shaping North–South collaboration and their links to epistemic injustice, the relatively small sample size limits the extent to which these results can be generalized across different contexts. Future research should expand the analytical scope by incorporating additional constructs and a larger sample size to generate more robust and generalizable findings. While including a broader range of constructs, such as institutional culture, funding mechanisms, power asymmetries, and communication dynamics, would allow for a more comprehensive understanding of how testimonial and hermeneutical injustice manifest in collaborative settings. At the same time, increasing the sample size across diverse geographic and institutional contexts would improve the statistical strength and reliability of the findings, enabling more concrete conclusions and stronger empirical grounding. Together, these steps would help refine and validate the current insights while deepening the analysis of epistemic injustice in North–South collaborations.

## Data Availability

The original contributions presented in the study are included in the article/Supplementary material, further inquiries can be directed to the corresponding author.
