# Supplementary material for: Determinants of collaboration dynamics and challenges in North-South data-driven health research in the African context: an epistemic injustice perspective
Source: Front Res Metr Anal. 2026 Sep 10;11:1885894. doi: 10.3389/frma.2026.1885894 (PMC13601277; doi:10.3389/frma.2026.1885894)
Supplement: Supplementary file 1 [file Data_Sheet_1.pdf]

APHREA-DST Survey on North-South Collaboration

Introduction

You are invited to participate in a research study examining the experiences and perceptions of researchers and practitioners involved in North-South collaborations in the areas of artificial intelligence (AI) and data science for health. As you may be aware, these partnerships hold great potential for strengthening healthcare systems across Africa. However, they also raise important questions about leadership roles, data ownership, capacity development, sustainability, and the recognition of local knowledge systems.

Conceived during a joint workshop hosted by the Brocher Foundation in Geneva, Switzerland, the study is condcuted by a team of researchers from Addis Ababa University (Ethiopia), Columbia University (USA), University of Oslo (Norway) and University of Nairobi (Kenya). It is part of a broader study designed by APHREA-DST seeking to understand how partners from the global south experience collaboration within various projects. The main focus is understanding how these collaborations reinforce or challenge patterns of epistemic injustice, and what strategies might contribute to more equitable and sustainable partnerships. Needless to say that your insights will help shape recommendations for more inclusive, locally grounded, and sustainable practices in international AI and data science collaborations in health.

The broader study has been reviewed by IRB at Addis Ababa University (063/21/SPH) where Addis Ababa University is responsible for keeping the data safe. Processing of data and presentation of findings will not identify you nor your responses.

Please note that the survey will take approximately 15–20 minutes to complete and participation is entirely voluntary. By submitting your responses, you consent to the storage and processing of your data for research purposes and to the publication of findings. All responses will be kept strictly confidential and will be deleted after the research publications are completed

If you have questions about the survey, please contact
Dr. Rahel Bekele: rahel.bekele@aau.edu.et,
Prof. Kiros Berhane: ktb2132@cumc.columbia.edu, or
Prof Anne Moen: anne.moen@medisin.uio.no

Thank you for your valuable contribution to this important research.

I. General Information

1. 1. Your primary institutional affiliation is based in:

Mark only one oval.

- ( ) Primarily Global North
- ( ) Primarily Global South

2. 2. Your role in the collaboration:

Check all that apply.

- [ ] Project leader / Principal coordinator
- [ ] Co-investigator
- [ ] Data Manager/Analyst
- [ ] Junior Researcher / Early Career Researcher
- [ ] Post Doctoral Researcher
- [ ] Other: ____________________

3. 3. Type of collaboration:

Check all that apply.

- [ ] Research project
- [ ] Development/Implementation project
- [ ] Capacity building/training
- [ ] Other: ____________________

4. 4. Years of experience in collaborative projects:

Mark only one oval.

- less than a year
- 1 to 2 years
- 3 to 5 years
- More than 5 years

5. 5. Number of North–South projects involved in:

Mark only one oval.

- Just 1
- 2 - 4
- More than 4

6. 6. Highest Level of education

Mark only one oval.

- Doctoral
- Masters
- Bachelors
- Other: ______________________

7. 7. Academic Rank

Mark only one oval.

- Professor
- Associate Professor
- Assistant Professor
- Lecturer
- Not in academia

II. Leadership and Collaboration

For each statement, select the option that best reflects your level of agreement or disagreement

8. 1. Leaders from the Global North are trusted as credible sources of knowledge

Mark only one oval.

	1	2	3	4	5	
Stro						Strongly Agree

9. 2. Leaders from the Global North facilitate mutual understanding across contexts and available knowledge

Mark only one oval.

	1	2	3	4	5	
Stro						Strongly Agree

10. 3. Local knowledge and experiences, community-based knowledge, indigenous approaches are elicited/ respected / integrated

Mark only one oval.

	1	2	3	4	5	
Stro	◯	◯	◯	◯	◯	Strongly Agree

11. 4. Leaders from the Global North recognize and challenge own potential biases in framing and interpreting knowledge.

Mark only one oval.

	1	2	3	4	5	
Stro	◯	◯	◯	◯	◯	Strongly Agree

Challenges related to leadeship and cooperation

For each item given below, please select the level of challenge that best reflects your experience

12. 5. Imbalance in funding control and financial decision-making

Mark only one oval.

	1	2	3	4	5	
Not	◯	◯	◯	◯	◯	Major challenge

13. 6. Perception that Global South leadership lacks the needed capacity

Mark only one oval.

	1	2	3	4	5	
Not	◯	◯	◯	◯	◯	Major challenge

14. 7. Partners from the Global North dominate in defining research questions, processes and outputs

Mark only one oval.

	1	2	3	4	5	
Not	◯	◯	◯	◯	◯	Major challenge

15. 8. Limited support for capacity development in leadership and governance

Mark only one oval.

	1	2	3	4	5	
Not	◯	◯	◯	◯	◯	Major challenge

16. 9. Lack of ethical collaboration

Mark only one oval.

	1	2	3	4	5	
Not	◯	◯	◯	◯	◯	Major challenge

III. Data Ownership

For each statement, select the option that best reflects your level of agreement or disagreement

17. 1. Data ownership structures allow partners from the global south to interpret and frame data in locally meaningful ways

Mark only one oval.

	1	2	3	4	5	
Stro	◯	◯	◯	◯	◯	Strongly Agree

18. 2. Communities from the Global South are involved in decisions about how their data is collected, interpreted, and used

Mark only one oval.

	1	2	3	4	5	
Stro	◯	◯	◯	◯	◯	Strongly Agree

19. 3. Partners from the Global North acknowledge historical imbalances in knowledge production.

Mark only one oval.

	1	2	3	4	5	
Stro	◯	◯	◯	◯	◯	Strongly Agree

20. 4. Partners from the global north address historical imbalances in data control.

Mark only one oval.

	1	2	3	4	5	
Stro	◯	◯	◯	◯	◯	Strongly Agree

21. 5. Data ownership arrangements prevent the extraction of knowledge without reciprocal benefits to partners from the Global South.

Mark only one oval.

	1	2	3	4	5	
Stro	◯	◯	◯	◯	◯	Strongly Agree

Challenges related to data ownership

For each item given below, please select the level of challenge that best reflects your experience

22. 6. Lack of clarity on data ownership at project start.

Mark only one oval.

	1	2	3	4	5	
Not	◯	◯	◯	◯	◯	Major challenge

23. 7. Insufficient infrastructure in the Global South to manage and safeguard data.

Mark only one oval.

	1	2	3	4	5	
Not	◯	◯	◯	◯	◯	Major challenge

24. 8. Power imbalances leading to Global North dominance in data decisions.

Mark only one oval.

	1	2	3	4	5	
Not	◯	◯	◯	◯	◯	Major challenge

25. 9. Limited participation from the Global South in interpreting and disseminating data.

Mark only one oval.

	1	2	3	4	5	
Not	◯	◯	◯	◯	◯	Major challenge

IV. Human Capacity Building

For each statement, select the option that best reflects your level of agreement or disagreement

26. 1. Building human capacity in the Global South supports the recognition of local knowledge and expertise.

Mark only one oval.

	1	2	3	4	5	
Stro	◯	◯	◯	◯	◯	Strongly Agree

27. 2. Capacity building enables partners from the Global South to frame and interpret knowledge in culturally relevant ways .

Mark only one oval.

	1	2	3	4	5	
Stro	◯	◯	◯	◯	◯	Strongly Agree

28. 3. Partners from the Global South have equal opportunities to contribute their perspectives in decision-making.

Mark only one oval.

	1	2	3	4	5	
Stro	◯	◯	◯	◯	◯	Strongly Agree

29. 4. Partners from the Global North actively challenge biases that undermine expertise from the Global South.

Mark only one oval.

	1	2	3	4	5	
Stro	◯	◯	◯	◯	◯	Strongly Agree

30. 5. Capacity development efforts address historical inequalities in knowledge production and leadership.

Mark only one oval.

	1	2	3	4	5	
Stro	◯	◯	◯	◯	◯	Strongly Agree

Challenges related to Human Capacity

For each item given below, please select the level of challenge that best reflects your experience

31. 6. Limited funding for AI / Data Science training activities.

Mark only one oval.

	1	2	3	4	5	
Not	◯	◯	◯	◯	◯	Major challenge

32. 7. Limited funding for research / development activities in general

Mark only one oval.

	1	2	3	4	5	
Not	◯	◯	◯	◯	◯	Major challenge

33. 8. Unequal access to training resources.

Mark only one oval.

	1	2	3	4	5	
Not	◯	◯	◯	◯	◯	Major challenge

34. 9. Lack of institutional support for capacity retention.

Mark only one oval.

	1	2	3	4	5	
Not	◯	◯	◯	◯	◯	Major challenge

V. Project Practice and Participation

For each statement, select the option that best reflects your level of agreement or disagreement

35. 1. Active participation of partners from the Global South ensures their knowledge and voices are respected.

Mark only one oval.

	1	2	3	4	5	
Stro	◯	◯	◯	◯	◯	Strongly Agree

36. 2. Partners from the Global South frame problems and interpret results in ways meaningful to their contexts.

Mark only one oval.

	1	2	3	4	5	
Stro	◯	◯	◯	◯	◯	Strongly Agree

37. 3. Partners from the Global South are active in framing and interpreting knowledge within their cultural contexts.

Mark only one oval.

	1	2	3	4	5	
Stro	◯	◯	◯	◯	◯	Strongly Agree

38. 4. Partners from the Global South are actively involved in shaping research questions and methodologies.

Mark only one oval.

	1	2	3	4	5	
Stro	◯	◯	◯	◯	◯	Strongly Agree

39. 5. The project fosters co-production of knowledge.

Mark only one oval.

	1	2	3	4	5	
Stro	◯	◯	◯	◯	◯	Strongly Agree

40. 6. Partners from the Global North encourage and facilitate equitable participation.

Mark only one oval.

	1	2	3	4	5	
Stro	◯	◯	◯	◯	◯	Strongly Agree

Challenges related to project practice and participation

For each item given below, please select the level of challenge that best reflects your experience

41. 7. Unequal participation in decision-making processes.

Mark only one oval.

	1	2	3	4	5	
Not	◯	◯	◯	◯	◯	Major challenge

42. 8. Insufficient mechanisms for conflict resolution.

Mark only one oval.

	1	2	3	4	5	
Not	◯	◯	◯	◯	◯	Major challenge

43. 9. Language and communication barriers hinder participation.

Mark only one oval.

	1	2	3	4	5	
Not	◯	◯	◯	◯	◯	Major challenge

44. 10. Cross-border mobility (e.g., visas, conference travel).

Mark only one oval.

	1	2	3	4	5	
Not	◯	◯	◯	◯	◯	Major challenge

45. 11. Reasonable funding (e.g., grants, stipends, research support).

Mark only one oval.

	1	2	3	4	5	
Not	◯	◯	◯	◯	◯	Major challenge

VI. Project Transition

For each statement, select the option that best reflects your level of agreement or disagreement

46. 1. Transition practices recognize knowledge and leadership of partners from the global south

Mark only one oval.

	1	2	3	4	5	
Stro	◯	◯	◯	◯	◯	Strongly Agree

47. 2. Partners from the global south are involved in interpreting and reframing knowledge during transitions.

Mark only one oval.

	1	2	3	4	5	
Stro	◯	◯	◯	◯	◯	Strongly Agree

48. 3. Transition processes actively address historical imbalances in decision-making and knowledge control.

Mark only one oval.

	1	2	3	4	5	
Stro	◯	◯	◯	◯	◯	Strongly Agree

49. 4. Transition planning includes measures to prevent loss of local knowledge and expertise.

Mark only one oval.

	1	2	3	4	5	
Stro	◯	◯	◯	◯	◯	Strongly Agree

Challenges related to project transition

For each item given below, please select the level of challenge that best reflects your experience

50. 5. Lack of clear transition plans or guidelines.

Mark only one oval.

	1	2	3	4	5	
Not	◯	◯	◯	◯	◯	Major challenge

51. 6. Power imbalances affecting who controls the transition process.

Mark only one oval.

	1	2	3	4	5	
Not	◯	◯	◯	◯	◯	Major challenge

52. 7. Loss of key personnel during transition phases.

Mark only one oval.

	1	2	3	4	5	
Not	◯	◯	◯	◯	◯	Major challenge

53. 8. Poor communication between partners during transitions.

Mark only one oval.

	1	2	3	4	5	
Not	◯	◯	◯	◯	◯	Major challenge

VII. Access to resources (human, data, infrastructure)

For each statement, select the option that best reflects your level of agreement or disagreement

54. 1. Limited access to data and infrastructure undermines expertise from the Global South.

Mark only one oval.

	1	2	3	4	5	
Stro	◯	◯	◯	◯	◯	Strongly Agree

55. 2. Resource inequities restrict ability of partners from the Global South to frame and interpret knowledge in locally meaningful ways

Mark only one oval.

	1	2	3	4	5	
Stro	◯	◯	◯	◯	◯	Strongly Agree

56. 3. Partners from the Global North actively work to mitigate resource inequalities

Mark only one oval.

	1	2	3	4	5	
Stro	◯	◯	◯	◯	◯	Strongly Agree

57. 4. Resource disparities reinforce historical patterns of marginalizing knowledge and perspectives of partners from the global south.

Mark only one oval.

	1	2	3	4	5	
Stro	◯	◯	◯	◯	◯	Strongly Agree

Challenges related to access to resources

For each item given below, please select the level of challenge that best reflects your experience

58. 5. Lack of funding for human resource development in the Global South.

Mark only one oval.

	1	2	3	4	5	
Not	◯	◯	◯	◯	◯	Major challenge

59. 6. Limited infrastructure and access in the Global South

Mark only one oval.

	1	2	3	4	5	
Not	◯	◯	◯	◯	◯	Major challenge

60. 7. Limited data access in the Global South.

Mark only one oval.

	1	2	3	4	5	
Not	◯	◯	◯	◯	◯	Major challenge

61. 8. Unequal distribution of resources favoring partners in the Global North

Mark only one oval.

	1	2	3	4	5	
Not	◯	◯	◯	◯	◯	Major challenge

VIII. Social and cultural practices

For each statement, select the option that best reflects your level of agreement or disagreement

62. 1. Cultural differences have led the knowledge of partners in the Global South to be undervalued

Mark only one oval.

	1	2	3	4	5	
Stro	◯	◯	◯	◯	◯	Strongly Agree

63. 2. Social and cultural differences affect ability of partners in the Global South to express their perspectives and interpret knowledge.

Mark only one oval.

	1	2	3	4	5	
Stro	◯	◯	◯	◯	◯	Strongly Agree

64. 3. Partners in the Global North recognize and actively address cultural barriers.

Mark only one oval.

	1	2	3	4	5	
Stro	◯	◯	◯	◯	◯	Strongly Agree

65. 4. Efforts are made to adapt knowledge production processes to cultural contexts of the Global South.

Mark only one oval.

	1	2	3	4	5	
Stro	◯	◯	◯	◯	◯	Strongly Agree

Challenges related to social and cultural practices

For each item given below, please select the level of challenge that best reflects your experience

66. 5. Differences in work ethics and time management styles.

Mark only one oval.

	1	2	3	4	5	
Not	◯	◯	◯	◯	◯	Major challenge

67. 6. Conflicting expectations around roles, responsibilities, and authority.

Mark only one oval.

	1	2	3	4	5	
Not	◯	◯	◯	◯	◯	Major challenge

68. 7. Social exclusion based on cultural differences.

Mark only one oval.

	1	2	3	4	5	
Not	◯	◯	◯	◯	◯	Major challenge

Thank you for your valuable time.

This content is neither created nor endorsed by Google.

Google Forms
