# Supplementary material for: Determinants of collaboration dynamics and challenges in North-South data-driven health research in the African context: an epistemic injustice perspective
Source: Front Res Metr Anal. 2026 Sep 10;11:1885894. doi: 10.3389/frma.2026.1885894 (PMC13601277; doi:10.3389/frma.2026.1885894)
Supplement: Supplementary file 2 [file Data_Sheet_2.pdf]

CFA

GL

lavaan 0.6-21 ended normally after 19 iterations		
Estimator	ML	
Optimization method	NLMINB	
Number of model parameters	14	
	Used	Total
Number of observations	103	109
Model Test User Model:		
Test statistic	39.148	
Degrees of freedom	14	
P-value (Chi-square)	0.000	
Model Test Baseline Model:		
Test statistic	324.223	
Degrees of freedom	21	
P-value	0.000	
User Model versus Baseline Model:		
Comparative Fit Index (CFI)	0.917	
Tucker-Lewis Index (TLI)	0.876	
Loglikelihood and Information Criteria:		
Loglikelihood user model (H0)	-973.573	
Loglikelihood unrestricted model (H1)	NA	
Akaike (AIC)	1975.145	
Bayesian (BIC)	2012.031	
Sample-size adjusted Bayesian (SABIC)	1967.808	
Root Mean Square Error of Approximation:		
RMSEA	0.132	
90 Percent confidence interval - lower	0.084	
90 Percent confidence interval - upper	0.182	
P-value H_0: RMSEA <= 0.050	0.004	
P-value H_0: RMSEA >= 0.080	0.962	

Standardized Root Mean Square Residual:

SRMR	0.058

Parameter Estimates:

Standard errors	Standard
Information	Expected
Information saturated (h1) model	Structured

Latent Variables:

	Estimate	Std.Err	z-value	P(>|z|)	Std.lv	Std.all
GL =~						
Ques2_5	1.000				0.864	0.690
Ques2_7	0.970	0.141	6.884	0.000	0.838	0.764
Ques2_8	0.706	0.125	5.641	0.000	0.610	0.613
Ques2_9	0.973	0.151	6.449	0.000	0.841	0.709
Ques3_6	0.932	0.150	6.215	0.000	0.805	0.681
Ques3_8	1.084	0.152	7.146	0.000	0.937	0.799
Ques3_9	0.828	0.139	5.953	0.000	0.715	0.650

Variances:

	Estimate	Std.Err	z-value	P(>|z|)	Std.lv	Std.all
.Ques2_5	0.822	0.131	6.270	0.000	0.822	0.524
.Ques2_7	0.500	0.087	5.767	0.000	0.500	0.416
.Ques2_8	0.617	0.094	6.578	0.000	0.617	0.624
.Ques2_9	0.698	0.113	6.163	0.000	0.698	0.497
.Ques3_6	0.749	0.119	6.313	0.000	0.749	0.536
.Ques3_8	0.497	0.092	5.403	0.000	0.497	0.362
.Ques3_9	0.701	0.109	6.449	0.000	0.701	0.578
GL	0.746	0.197	3.794	0.000	1.000	1.000

HC

lavaan 0.6-21 ended normally after 14 iterations

Estimator	ML	
Optimization method	NLMINB	
Number of model parameters	4	
	Used	Total
Number of observations	98	109

Model Test User Model:

Test statistic	6.089
Degrees of freedom	2
P-value (Chi-square)	0.048
Model Test Baseline Model:	
Test statistic	89.655
Degrees of freedom	3
P-value	0.000
User Model versus Baseline Model:	
Comparative Fit Index (CFI)	0.953
Tucker-Lewis Index (TLI)	0.929
Loglikelihood and Information Criteria:	
Loglikelihood user model (H0)	-383.562
Loglikelihood unrestricted model (H1)	NA
Akaike (AIC)	775.123
Bayesian (BIC)	785.463
Sample-size adjusted Bayesian (SABIC)	772.832
Root Mean Square Error of Approximation:	
RMSEA	0.144
90 Percent confidence interval - lower	0.013
90 Percent confidence interval - upper	0.282
P-value H_0: RMSEA <= 0.050	0.085
P-value H_0: RMSEA >= 0.080	0.849
Standardized Root Mean Square Residual:	
SRMR	0.146
Parameter Estimates:	
Standard errors	Standard
Information	Expected
Information saturated (h1) model	Structured
Latent Variables:	
Estimate Std.Err z-value P(>|z|) Std.lv Std.all	

HSC =~						
Ques4_6	1.000				0.779	0.843
Ques4_8	1.000				0.779	0.819
Ques8_6	1.000				0.779	0.591

Variances:						
	Estimate	Std.Err	z-value	P(>|z|)	Std.lv	Std.all
.Ques4_6	0.248	0.064	3.858	0.000	0.248	0.290
.Ques4_8	0.298	0.069	4.332	0.000	0.298	0.329
.Ques8_6	1.131	0.178	6.359	0.000	1.131	0.651
HSC	0.608	0.105	5.774	0.000	1.000	1.000

PIP		
lavaan 0.6-21 ended normally after 21 iterations		
Estimator	ML	
Optimization method	NLMINB	
Number of model parameters	10	
	Used	Total
Number of observations	95	109
Model Test User Model:		
Test statistic	18.370	
Degrees of freedom	5	
P-value (Chi-square)	0.003	
Model Test Baseline Model:		
Test statistic	288.216	
Degrees of freedom	10	
P-value	0.000	
User Model versus Baseline Model:		
Comparative Fit Index (CFI)	0.952	
Tucker-Lewis Index (TLI)	0.904	
Loglikelihood and Information Criteria:		
Loglikelihood user model (H0)	-547.404	
Loglikelihood unrestricted model (H1)	NA	

```
  Akaike (AIC)                                  1114.808
  Bayesian (BIC)                                1140.347
  Sample-size adjusted Bayesian (SABIC)         1108.775

Root Mean Square Error of Approximation:

  RMSEA                                            0.168
  90 Percent confidence interval - lower           0.090
  90 Percent confidence interval - upper           0.253
  P-value H_0: RMSEA <= 0.050                      0.010
  P-value H_0: RMSEA >= 0.080                      0.966

Standardized Root Mean Square Residual:

  SRMR                                             0.037

Parameter Estimates:

  Standard errors                               Standard
  Information                                   Expected
  Information saturated (h1) model            Structured

Latent Variables:
                   Estimate  Std.Err  z-value  P(>|z|)   Std.lv  Std.all
  PIP =~
    Ques6_5           1.000                               0.589    0.675
    Ques6_6           1.545    0.195    7.920    0.000    0.910    0.937
    Ques6_7           1.171    0.204    5.731    0.000    0.690    0.636
    Ques6_8           1.697    0.221    7.674    0.000    0.999    0.887
    Ques5_8           1.294    0.201    6.433    0.000    0.762    0.722

Variances:
                   Estimate  Std.Err  z-value  P(>|z|)   Std.lv  Std.all
   .Ques6_5           0.415    0.064    6.447    0.000    0.415    0.545
   .Ques6_6           0.115    0.040    2.886    0.004    0.115    0.122
   .Ques6_7           0.701    0.107    6.533    0.000    0.701    0.596
   .Ques6_8           0.269    0.060    4.509    0.000    0.269    0.212
   .Ques5_8           0.532    0.084    6.302    0.000    0.532    0.478
    PIP               0.347    0.095    3.633    0.000    1.000    1.000
```

Final model

```
lavaan 0.6-21 ended normally after 58 iterations

  Estimator                                         ML
```

Optimization method	NLMINB	
Number of model parameters	41	
	Used	Total
Number of observations	107	109
Number of missing patterns	4	
Model Test User Model:		
	Standard	Scaled
Test Statistic	115.565	102.920
Degrees of freedom	49	49
P-value (Chi-square)	0.000	0.000
Scaling correction factor		1.123
Yuan-Bentler correction (Mplus variant)		
Model Test Baseline Model:		
Test statistic	758.651	637.895
Degrees of freedom	66	66
P-value	0.000	0.000
Scaling correction factor		1.189
User Model versus Baseline Model:		
Comparative Fit Index (CFI)	0.904	0.906
Tucker-Lewis Index (TLI)	0.871	0.873
Robust Comparative Fit Index (CFI)		0.914
Robust Tucker-Lewis Index (TLI)		0.884
Loglikelihood and Information Criteria:		
Loglikelihood user model (H0)	-1457.936	-1457.936
Scaling correction factor		1.151
for the MLR correction		
Loglikelihood unrestricted model (H1)	NA	NA
Scaling correction factor		1.136
for the MLR correction		
Akaike (AIC)	2997.871	2997.871
Bayesian (BIC)	3107.457	3107.457
Sample-size adjusted Bayesian (SABIC)	2977.916	2977.916
Root Mean Square Error of Approximation:		

RMSEA				0.113	0.101	
90 Percent confidence interval - lower				0.086	0.075	
90 Percent confidence interval - upper				0.139	0.127	
P-value H_0: RMSEA <= 0.050				0.000	0.001	
P-value H_0: RMSEA >= 0.080				0.977	0.915	
Robust RMSEA					0.108	
90 Percent confidence interval - lower					0.077	
90 Percent confidence interval - upper					0.139	
P-value H_0: Robust RMSEA <= 0.050					0.002	
P-value H_0: Robust RMSEA >= 0.080					0.933	
Standardized Root Mean Square Residual:						
SRMR				0.066	0.066	
Parameter Estimates:						
Standard errors				Sandwich		
Information bread				Observed		
Observed information based on				Hessian		
Latent Variables:						
	Estimate	Std.Err	z-value	P(>|z|)	Std.lv	Std.all
NSC =~						
Ques4_1	1.000				0.581	0.594
Ques5_4	1.363	0.190	7.160	0.000	0.792	0.843
Ques5_5	1.279	0.192	6.667	0.000	0.743	0.950
GL =~						
Ques2_7	1.000				0.787	0.729
Ques2_9	1.111	0.162	6.875	0.000	0.875	0.746
Ques3_6	1.091	0.148	7.368	0.000	0.859	0.735
Ques3_8	1.097	0.136	8.089	0.000	0.863	0.749
HC =~						
Ques4_6	1.000				0.810	0.878
Ques4_8	1.000				0.810	0.827
PIP =~						
Ques6_6	1.000				0.951	0.966
Ques6_8	0.972	0.074	13.080	0.000	0.924	0.843
Ques5_8	0.792	0.114	6.958	0.000	0.754	0.703
Regressions:						
	Estimate	Std.Err	z-value	P(>|z|)	Std.lv	Std.all
NSC ~						
GL	0.281	0.139	2.029	0.042	0.381	0.381

HC	0.335	0.127	2.646	0.008	0.467	0.467
PIP	-0.284	0.117	-2.417	0.016	-0.464	-0.464
Covariances:						
	Estimate	Std.Err	z-value	P(>|z|)	Std.lv	Std.all
GL ~~						
HC	0.310	0.096	3.251	0.001	0.487	0.487
PIP	0.535	0.147	3.641	0.000	0.715	0.715
HC ~~						
PIP	0.340	0.099	3.442	0.001	0.441	0.441
Intercepts:						
	Estimate	Std.Err	z-value	P(>|z|)	Std.lv	Std.all
.Ques4_1	4.264	0.099	43.009	0.000	4.264	4.361
.Ques5_4	3.922	0.095	41.260	0.000	3.922	4.174
.Ques5_5	4.153	0.080	51.801	0.000	4.153	5.306
.Ques2_7	3.514	0.104	33.667	0.000	3.514	3.255
.Ques2_9	2.766	0.113	24.400	0.000	2.766	2.359
.Ques3_6	3.252	0.113	28.782	0.000	3.252	2.782
.Ques3_8	3.561	0.112	31.933	0.000	3.561	3.087
.Ques4_6	4.153	0.094	44.149	0.000	4.153	4.501
.Ques4_8	4.193	0.095	43.997	0.000	4.193	4.282
.Ques6_6	3.554	0.095	37.275	0.000	3.554	3.610
.Ques6_8	3.423	0.108	31.746	0.000	3.423	3.123
.Ques5_8	3.192	0.104	30.556	0.000	3.192	2.979
Variances:						
	Estimate	Std.Err	z-value	P(>|z|)	Std.lv	Std.all
.Ques4_1	0.618	0.116	5.333	0.000	0.618	0.647
.Ques5_4	0.256	0.091	2.799	0.005	0.256	0.290
.Ques5_5	0.060	0.053	1.137	0.256	0.060	0.098
.Ques2_7	0.546	0.107	5.084	0.000	0.546	0.468
.Ques2_9	0.610	0.120	5.067	0.000	0.610	0.443
.Ques3_6	0.629	0.119	5.295	0.000	0.629	0.460
.Ques3_8	0.585	0.094	6.255	0.000	0.585	0.440
.Ques4_6	0.195	0.057	3.410	0.001	0.195	0.230
.Ques4_8	0.303	0.089	3.397	0.001	0.303	0.316
.Ques6_6	0.064	0.053	1.209	0.227	0.064	0.066
.Ques6_8	0.347	0.060	5.832	0.000	0.347	0.289
.Ques5_8	0.580	0.126	4.615	0.000	0.580	0.505
.NSC	0.234	0.076	3.081	0.002	0.692	0.692
GL	0.620	0.160	3.870	0.000	1.000	1.000
HC	0.656	0.114	5.735	0.000	1.000	1.000
PIP	0.905	0.161	5.638	0.000	1.000	1.000
